# Pharmacokinetics and pharmacodynamics of enrofloxacin treatment of *Escherichia coli* in a murine thigh infection modeling

**DOI:** 10.1186/s12917-021-02908-8

**Published:** 2021-06-09

**Authors:** Xuesong Liu, Qingwen Yang, Yuying Fan, Yuanyi Du, Lei Lei, Dong Wang, Yun Liu

**Affiliations:** 1 Department of Veterinary Surgery, College of Veterinary Medicine, Heilongjiang Key Laboratory for Laboratory Animals and Comparative Medicine, Northeast Agricultural University, Harbin, 150030 China; 2 Laboratory of Veterinary Pharmacology, Branch of Animal Husbandry and Veterinary of Heilongjiang Academy of Agricultural Sciences, Qiqihar, China; 3 Laboratory of Veterinary Pharmacology, Department of Animal Science and Technology, Chongqing Three Gorges Vocational College, Chongqing, China

**Keywords:** Enrofloxacin, Neutropenic thigh infection model, *Escherichia coli*, Mice, Pharmacokinetic/Pharmacodynamic integration model

## Abstract

**Background:**

Enrofloxacin is an antibacterial drug with broad-spectrum activity that is widely indicated for veterinary use. We aim to develop the clinical applications of Enrofloxacin against colibacillosis by using the neutropenic mice thigh infection model.

**Results:**

The minimum inhibitory concentration (MIC) distribution of 67 isolated *E. coli* strains to ENR was calculated using CLSI guidelines. Whereas, the MIC_50_ value calculation was considered as the population PD parameter for ENR against *E. coli* strains. The MIC values of 15 *E. coli* strains were found to be nearest to the MIC_50_ i.e.*,* 0.25 μg/mL. Of all the tested strains, the PK-PD and *E. coli* disease model was established via selected *E. coli* strain i.e.*,* Heilong 15. We analyzed the PK characteristics of ENR and its metabolite ciprofloxacin (CIP) following a single subcutaneous (s.c.) injection of ENR (1.25, 2.5, 5, 10 mg/kg). The concentration-time profiling of ENR within the plasma specimens was determined by considering the non-compartmental analysis (NCA). The basic PK parameters of ENR for the peak drug concentration (C_max_) and the area under the concentration-time curve (AUC) values were found to be in the range of 0.27–1.97 μg/mL and 0.62–3.14 μg.h/mL, respectively. Multiple s.c. injection over 24 h (1.25, 2.5, 5, 10 mg/kg at various time points i.e.*,* 6, 8, 12, and 24 h respectively) were administered to assess the targeted PD values. The Akaike Information Criterion (AIC) was used to choose PD models, and the model with the lowest AIC was chosen. The inhibitory E_max_ model was employed to calculate the related PK-PD parameters. The results of our study indicated that there was a strong correlation between the AUC/MIC and various antibacterial activities (R^2^ = 0.9928). The target values of dividing AUC/MIC by 24 h for bacteriostatic action were 1-log10 reduction, 2-log10 reduction, and 3-log10 reduction 0.325, 0.4375, 0.63, and 0.95 accordingly.

**Conclusion:**

The identified pharmacodynamics targets for various antibacterial effects will be crucial in enhancing ENR clinical applications and serving as a key step in reducing bacterial resistance.

## Background

*Escherichia coli (E. coli)* is a Gram-negative bacterium that is responsible for a variety of animal diseases, including septicemia, enterocolitis, and diarrhea [1, 2]. Colibacillosis is an infectious syndrome caused by pathogenic *E. coli*, that has been linked to high rates of mortality and morbidity around the world, as well as significant losses in the poultry and livestock industries.

ENR is an effective broad-spectrum antibiotic, and is generally used against a wide range of pathogenic bacteria. It can be particularly used in the livestock and poultry industries to combat a variety of infectious diseases caused by *E. coli*. ENR is a third-generation fluoroquinolone widely used in veterinary medicines as an antimicrobial drug [3]. It is evident from the study that gram-negative bacteria appeared to be more sensitive to ENR treatment. However, fluoroquinolone resistance is emerging at an alarming level and because of this problem, the PK-PD model was used to design a reasonable drug dosage regimen. Thus, several approaches have been recommended, including the use of drug combinations to significantly enhance the antibacterial effects [4].

PK-PD modeling has been a significant strategy for providing an optimal dose regimen to prevent resistance problems [5]. The model can establish the relationship between drug concentration, antimicrobial effect, and time. Based on the scientific calculation, the model can provide the optimal dose regimen and dose interval [6]. Certain PK parameters (i.e., AUC and C_max_) and PD parameters (i.e., minimal inhibitory concentration (MIC) and time-kill curve) are commonly considered to integrate the PK-PD model. PK-PD indexes comprised the ratio of AUC divided by the MIC (AUC/MIC). The percentage time when exceeded from the drug MIC (%T > MIC) and the ratio was obtained from the peak concentration divided by the MIC (C_max_/MIC). However, when using the PK-PD index AUC/MIC values for the evaluation of breakpoints, the AUC/MIC has a time dimension (h) that makes it difficult for readers to comprehend and scientists to discuss. On the other hand, the scalar obtained by dividing the AUC/MIC by time interval gives values that are easier to understand. Moreover, dividing the AUC/MIC by time interval allows the assessment of a truly dimensionless ratio, and the computed numerical value has also directed clinical interpretation [7]. Based on the antibacterial effect of drugs, they can be categorized into concentration-dependent and time-dependent drugs. In the case of concentration-dependent drugs, fluoroquinolones are the more prominent, and their AUC/MIC and C_max_/MIC ratios comparatively indicated significant PK-PD indexes, as compared to %T > MIC for the antibacterial activity [8, 9].

The correlation between the host, pathogens, and the drug is usually determined via the neutropenic mice thigh infection model [10, 11]. The stability, maturity, and avoidance of the host immune system’s impact on the antibacterial effect are all major advantages of this model. In this context, Ferran et al., evaluated the resistance of marbofloxacin (fluoroquinolones) in *E. coli* strains by using the neutropenic mice thigh model of bacterial infection [12]. However, the in-vivo study of the combined effect of PK and PD to test ENR antibacterial effect against *E. coli* in the thigh model of bacterial infection in neutropenic mice has never been studied before.

Herein, we adopted the neutropenic mice thigh model of bacterial infection for the selection of the PK-PD index, which significantly correlates with the ENR potency against *E. coli*. In this study, the PD targets for various antibacterial effects were also evaluated. It has been suggested that these parameters can be employed with MIC_90_ data to cure a diversity of *E. coli* infections and to obtain a rational dosage schedule for optimizing the efficacy of both clinical and simulated treatments.

## Results

### In-vitro susceptibility testing

**Fig. 1:**
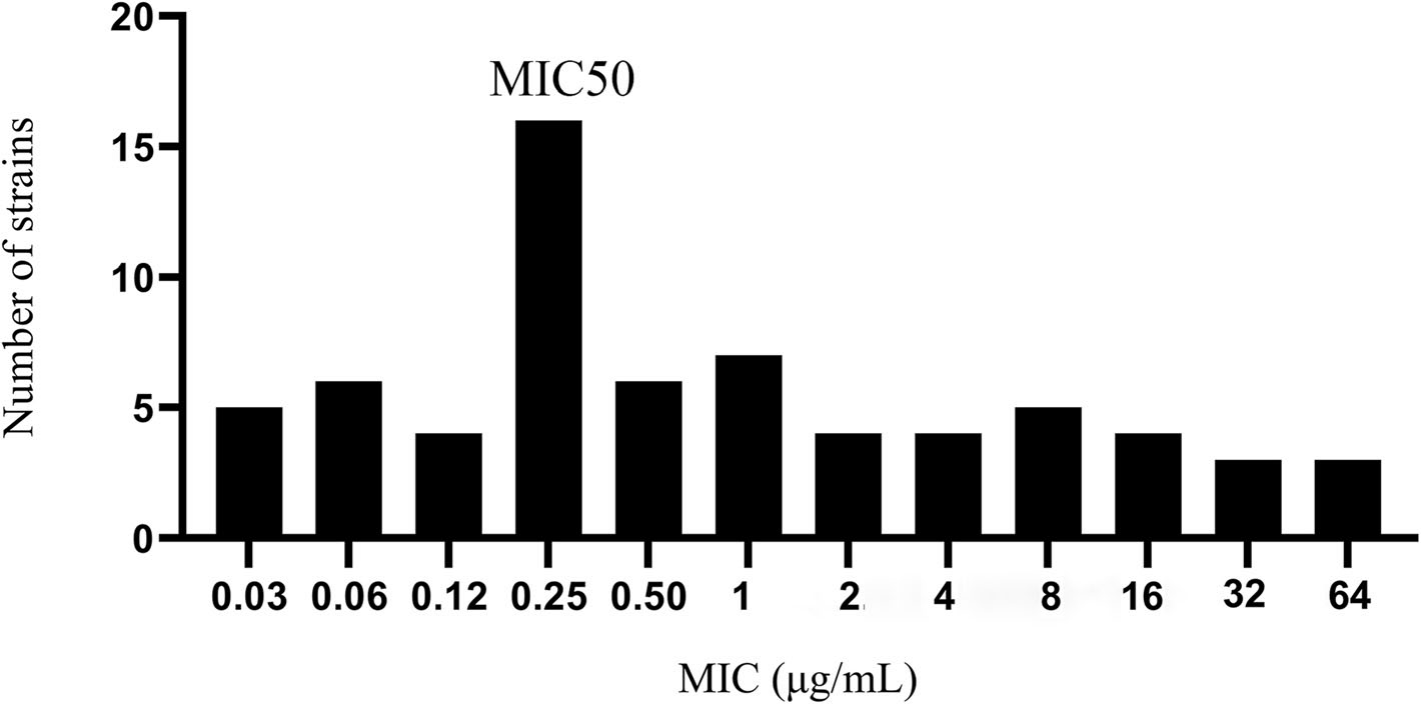
ENR MIC distribution of 67 *Escherichia coli* strains. On the X-axis, MIC values were given while Y-axis represents strain number in all MIC values.

The MIC distribution of 67 *E. coli* strains to ENR tested in mice plasma is depicted in Fig. 1. The MIC_50_ for ENR was found to be 0.25 μg/mL. The MIC values in MHB were approximately 1.7 times higher than those MIC values tested in mice plasma. To the designated *E. coli* strain Heilong 15, MIC values in MHB and mice plasma were 0.43 μg/mL and 0.25 μg/mL, respectively. The MIC values in MHB and mice plasma of 15 *E. coli* strains which were found close to the MIC_50_ are shown in Table. 1.

**Table 1:** The minimum inhibitory concentration (MIC) of Enrofloxacin against *E. coli* in Mueller-Hinton broth (MHB) and mice plasma

*E. coli* strain	MHB (μg/mL)	Mice plasma (μg/mL)	MHB/mice plasma ratio
Heilong 01	0.43	0.25	1.72
Heilong 02	0.5	0.22	2.27
Heilong 03	0.5	0.25	2
Heilong 04	0.25	0.22	1.16
Heilong 05	0.43	0.25	1.72
Heilong 06	0.5	0.22	2.27
Heilong 07	0.43	0.25	1.72
Heilong 08	0.25	0.22	1.13
Heilong 09	0.87	0.25	3.48
Heilong 10	0.25	0.22	1.13
Heilong 11	0.43	0.25	1.72
Heilong 12	0.25	0.22	1.13
Heilong 13	0.5	0.25	2
Heilong 14	0.25	0.22	1.16
Heilong 15	0.43	0.25	1.72
Average	0.418	0.236	1.76

### PK of ENR and CIP in neutropenic thigh infected mice

**Fig. 2:**
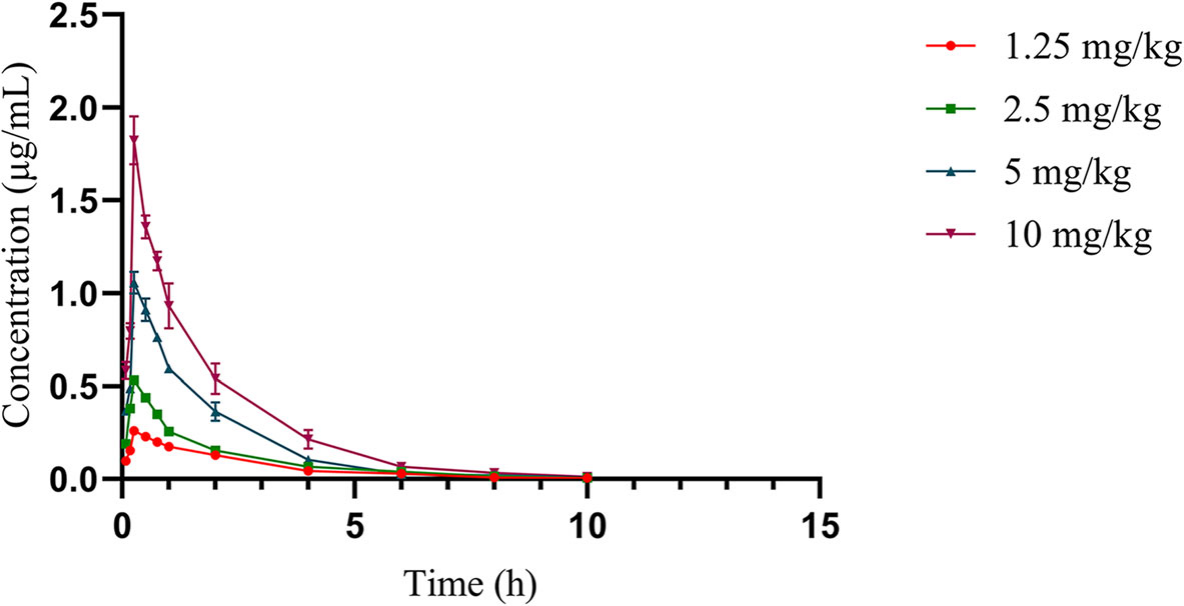
The ENR concentration-time course following single s.c injection of 1.25, 2.5, 5, and 10 mg/kg

The time courses of the mean plasma ENR concentrations in neutropenic infected mice following a single dose of 1.25, 2.5, 5, and 10 mg/kg (injected subcutaneously), are depicted in Fig. 2, while Table. 2 represents the concentration of CIP. The primary PK parameters of ENR are shown in Table. 3. The AUC and C_max_ values of ENR were 0.62 ~ 3.14 μg.h/mL and 0.27 ~ 1.97 μg/mL, respectively. The elevation was observed in AUC and C_max_ values with increasing dose concentration (1.25–10 mg/kg), while the range of T_1/2e_ values was from 1.35 to 1.65 h. Post 10 h, the ENR concentration in plasma was decreased below the detection limit, so, its detection could not be possible.

**Table 2:** The CIP concentration (μg/mL) in neutropenic infected mice following single s.c injection

Time (h)	Dosage regimen (mg/kg)			
	1.25	2.5	5	10
0.083	–	–	–	–
0.167	–	0.029 ± 0.009	0.031 ± 0.011	0.037 ± 0.014
0.25	0.019 ± 0.007	0.037 ± 0.011	0.057 ± 0.019	0.09 ± 0.027
0.5	0.009 ± 0.002	0.019 ± 0.004	0.026 ± 0.009	0.056 ± 0.017
0.75	–	0.007 ± 0.002	0.009 ± 0.002	0.029 ± 0.009
1	–	–	–	0.017 ± 0.004
2	–	–	–	0.006 ± 0.001

**Table 3:** The Pharmacokinetic parameters of ENR in neutropenic thigh infected mice plasma

Parameter (units)	Does			
	1.25	2.5	5	10
T1/2e (h)	1.65	1.61	1.32	1.37
T_max_ (h)	0.25	0.25	0.18	0.25
AUC (μg.h/mL)	0.62	0.96	1.92	3.14
C_max_ (μg/mL)	0.27	0.51	1.06	1.97
MRT (h)	1.57	2.08	2.13	2.29
Vss (L/kg)	4.55	5.06	5.58	6.68
CL (L/h.kg)	2.71	2.66	2.47	2.93
AUMC (μg.h^2^/mL)	0.72	1.94	4.64	7.27

C_max_, maximum concentration in plasma; T_max_ time to achieve maximum concentration; T1/2e, elimination half-life; AUC, area under the concentration-time curve; MRT, mean residence time; Vss, volume of distribution; CL, systemic clearance; AUMC, area under the first moment-time curve

### In-vitro antimicrobial activity

**Fig. 3:**
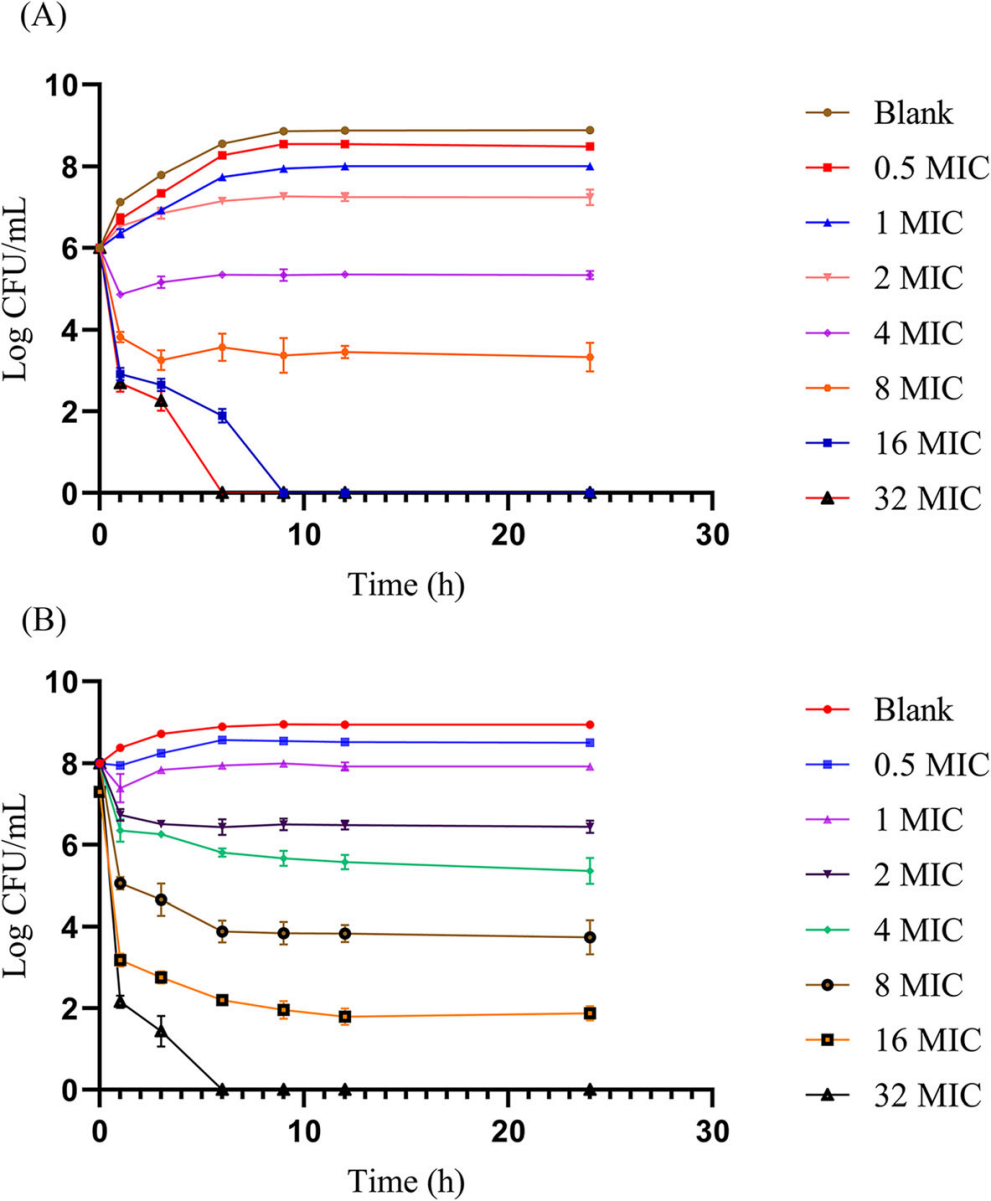
The in-vitro killing curve of ENR against *E. coli* (Heilong 15). (**A**). Antibacterial effect starting with a bacterial density of 10^6^ CFU/mL (**B**) Antibacterial effects starting with a bacterial density of 10^8^ CFU/mL

The in-vitro killing curve which indicates that ENR is a drug that depends on concentration is depicted in Fig. 3. When exposed to the increased concentration of ENR (16 MIC or 32 MIC), the bacterial CFUs were considerably reduced to unnoticeable levels (<10 CFU). As the concentration reached > 4 × MIC, ENR reached the level where it inhibits the bacterial growth, while the killing of bacteria was found to be at <3log CFU/mL.

### PK-PD integration and modeling

**Fig. 4:**
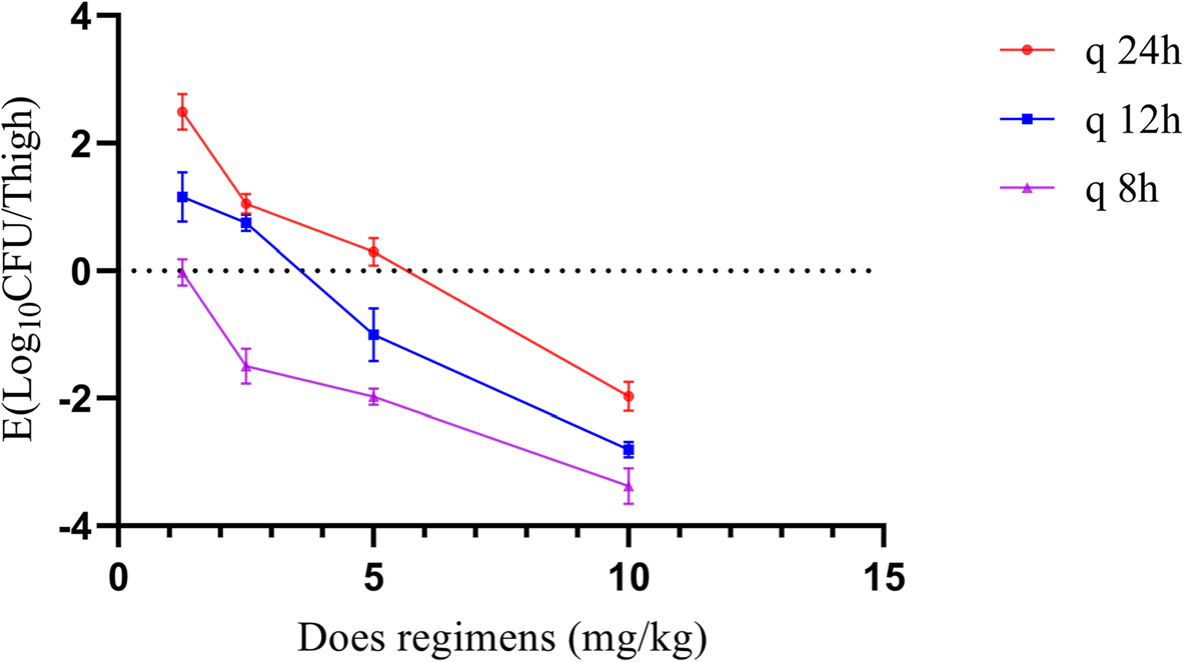
In-vivo ENR PD studies via murine neutropenic thigh infection model. Each symbol shows the mean value of thigh bacterial infection from five infected mice (*Escherichia coli* Heilong 15). Twelve dose regimens of ENR were used to treat the *E. coli* Heilong 15 infection. The variations in the log10 number of colony-forming unit (CFU)/thigh were evaluated in the beginning and post 24 hrs of treatment. Data points under the horizontal dashed line indicate killing and points above the horizontal dashed line showed growth and development

**Fig. 5:**
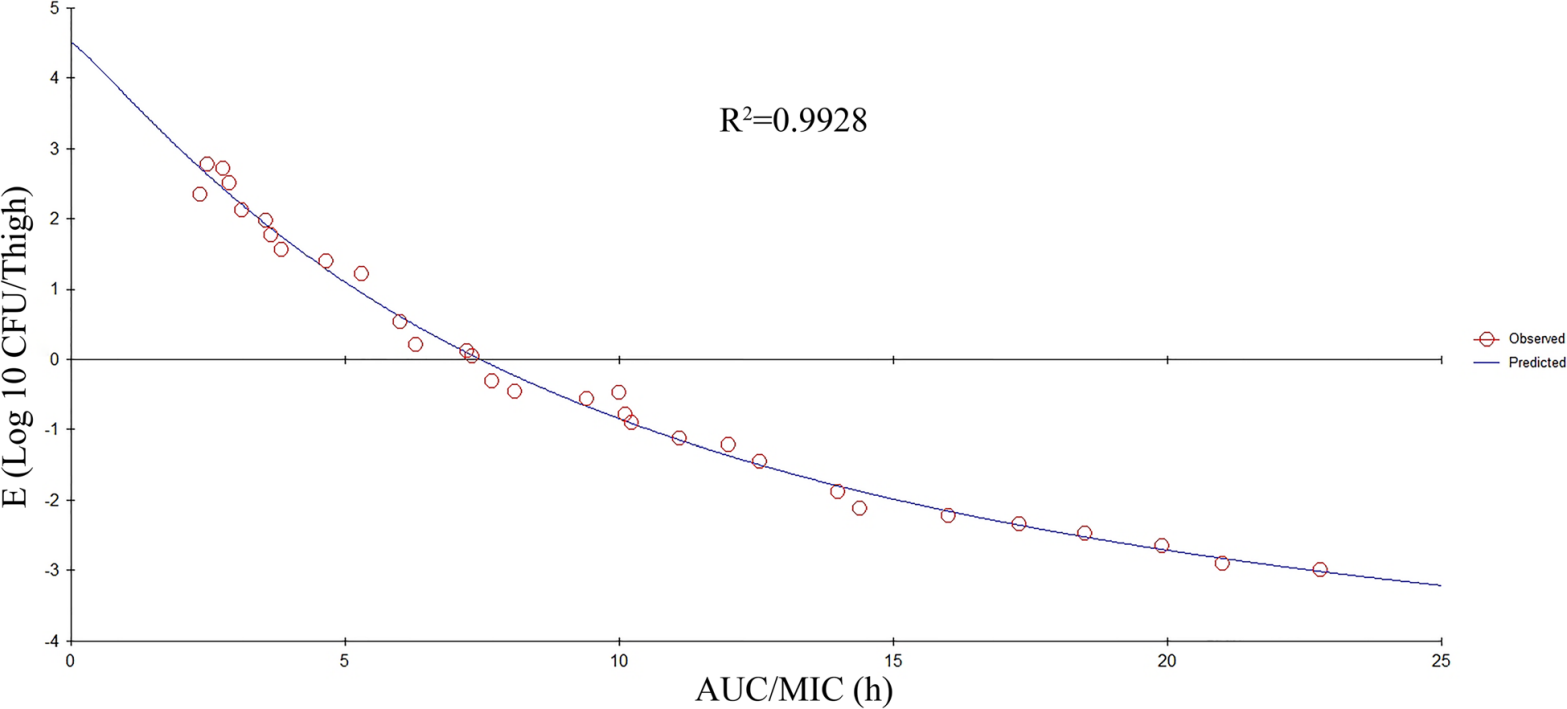
Relationship between AUC/MIC and E. E is defined as the reduction of bacterial count post 24 hrs of treatment. R^2^ represents the correlation coefficient

**Fig. 6:**
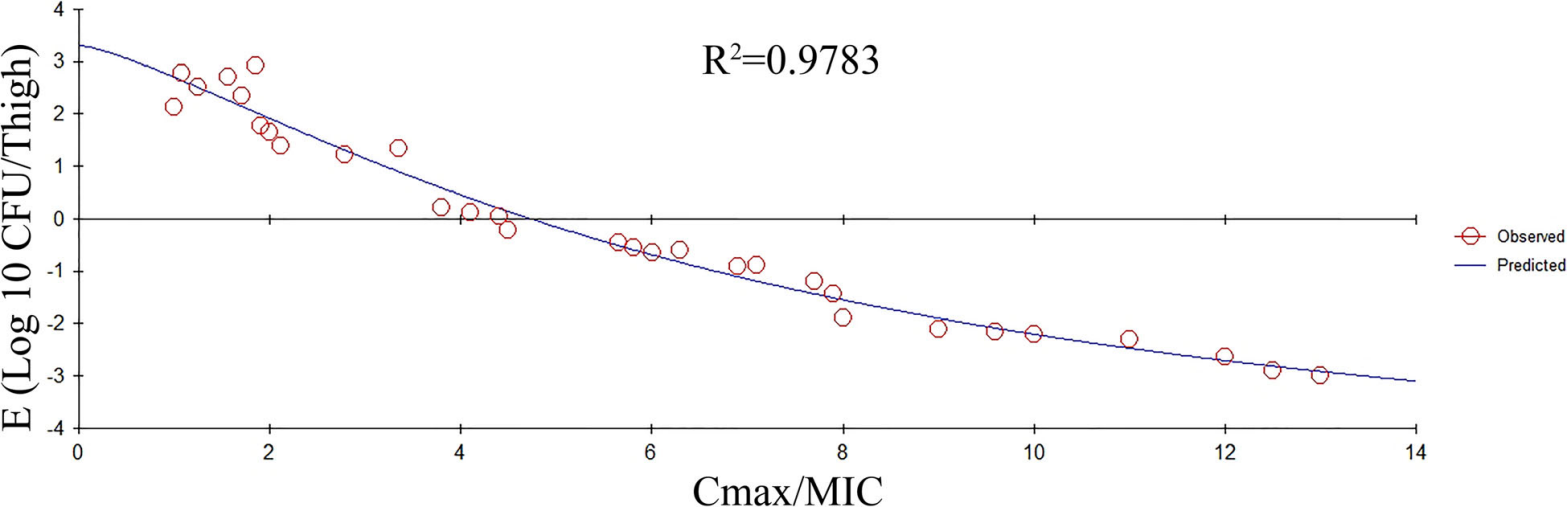
Relationship between C_max_/MIC and E. E is defined as the reduction of bacterial count post 24 hrs of treatment. R^2^ represents the correlation coefficient

At the beginning of the therapy, mice had 5.35 log10 CFU/ thigh almost 4 h post-inoculation. In the control group (untreated), the bacterial capacity reached 7.55 log10 CFU/thigh across the overnight treatment. The increased antibacterial effects in the experimental group were decreased to a rate of 2.67 ± 0.25 per thigh 24 h post-treatment. The antibacterial effects are indicated in Fig. 4. The key PK/PD parameters are given in Table. 4. In this model, the AUC/MIC was considered as the best PK-PD Index of the antibacterial effect (R^2^ = 0.9928). The association between the PK-PD Index and the antibacterial effect is indicated in Figs. 5 and 6. The bacteriostatic action, 1-log10 reduction, 2-log10 reduction, and 3- log 10 reductions were obtained when the dividing AUC/MIC by 24 h reached 0.325, 0.4375, 0.63, and 0.95, accordingly.

**Table 4:** The PK-PD parameter estimates for the dividing AUC0–24/MIC by 24 h to obtain different antibacterial effects

Parameter	Values
Emax (log10 CFU/thigh)	3.86
E0 (log10 CFU/thigh)	−4.95
EC50 (h)	9.05
N	1.38
Dividing AUC0–24/MIC by 24 h for bacteriostatic action	0.325
Dividing AUC0–24/MIC by 24 h for 1-log10 reduction	0.4375
Dividing AUC0–24/MIC by 24 h for 2-log10 reduction	0.63
Dividing AUC0–24/MIC by 24 h for 3-log10 reduction	0.95

E_max_ is △logCFU24 h in the control sample (drug-free); E0 is △logCFU24 hrs in the experimental sample comprising ENR when the maximum potential against bacterial growth was obtained; EC50 represents the PK-PD index for the drug which shows 50% of the highest antibacterial effect; N represents Hill coefficient (demonstrating the steepness of the effect curve obtained from PK-PD index)

### Estimation of doses

The doses estimated for dairy cattle, steer, and pig are shown in Table. 5**.**

**Table 5:** Estimated doses for dairy cattle, steer and pig

Animal species	Conversion rate	fu	Clearance	F	1-log10 reduction MIC_90_ = 0.25	3-log10 reduction MIC_90_ = 0.25
Dairy cattle	59%	40.6%	34.8 L/day/kg	1	4.05 mg/kg	11.1 mg/kg
Steer	64%	39.2%	16.8 L/day/kg	1	1.42 mg/kg	6.78 mg/kg
Pig	10%	66%	4.43 L/day/kg	1	0.66 mg/kg	1.45 mg/kg

The conversion rate is the ENR to CIP. fu is the free (unbound) fraction. F is the absolute bioavailability. MIC_90_ is the 90th percentile of MIC distribution

## Discussion

The PK studies of ENR and its metabolites have been carried out in various animals, such as broilers, foals, goats, calves, dairy cows, swine, goats, and buffalo calves [17, 19, 21, 23–25]. In the present study, the mean value of ENR T_1/2e_ in plasma was 1.48 h in mice post single injection (s.c.), which is almost similar to the previous studies [26, 27]. However, the T1/2e value in the existing study was considerably lower than foals (17.10 h), dairy cows (3.69 h), beef steers (5.15 h), swine (6.69 h), and buffalo calves (5.14 h) [17, 19, 21, 23]. In the existing research work, the C_max_ value in the neutropenic mice (at 2.5 mg/kg dose) was 0.27 μg/m and was found to be lower as compared with the swine (1.09 μg/mL) [19]. At 5 mg/kg dose, the value was 0.51 μg/mL that was considerably lower than the value in the goat (4.30 μg/mL) [25]. The AUC of this study at the dosage of 10 mg/kg was found to be 3.14 μg.h/mL that was considerably decreased than the AUC of turkey (23.76 μg.h/mL) at the same dose [28]. The leading cause of variations in the values may be due to diverse animal species. In the present study, the dose was proportional to both the AUC and C_max_ values. The metabolite conversion ratio of CIP was 5–10%, which showed similarity to the earlier studies [27, 29]. However, the metabolic conversion ratio in mice was found to be considerably lower than that in the dairy cow (59%), in beef steers (64%), and buffalo calves (47%) [17, 21]. Similar observations have also been found in other PK / PD models of ENR [19, 24]. In various types of animals, the rate of conversion of ENR to CIP is relatively different. Ciprofloxacin, a metabolite of ENR, has at least the same antibacterial effect as ENR [30]. In particular, the conversion rate should be taken into account in the calculation of dosage. If we just consider ENR’s antibacterial effect and disregard Ciprofloxacin’s antibacterial effect, the measured dose would be too high, which is not good for animal health, particularly in animal species with high conversion rates like dairy cows and steers.

Previous studies have suggested that the higher MIC value calculated in MHB than in similar biological matrices might be artificially elevated [31, 32]. As a result, the MIC from biological matrix samples is needed rather than MHB. In this study, the values of MIC in MHB were found to be higher (1.7 times) than the MIC values in mice plasma. In the previous study, the MIC values tested in MHB were reported 8 times higher than those tested in mice serum [33]. Hence, it is necessary to measure the values of MIC in the corresponding biological matrix that provides a guarantee for the integrated PK-PD model in the following step. The larger MIC values caused via the measured liquid matrix will lead to the relatively smaller PK-PD indexes. As a result, PD target values will be lower which tends to decrease dosage by calculating.

PK-PD modeling is an effective tool in the veterinary field which explores the impact of drugs against microbes and, hence, it is recommended to be used in the preclinical and clinical development of veterinary drugs for optimizing the dosing approach [5, 8]. The selection of a highly effective PK/PD index is regarded as an important step. Fluoroquinolone is mainly categorized as an antimicrobial drug that is dependent on concentration [34]. In the earlier studies, the PK-PD index AUC/MIC and C_max_/MIC indicated a strong association with the activities against bacteria [35]. In this study, through the in-vitro killing curve and in-vivo PD studies, ENR exhibited classic concentration-dependent characteristics. The correlation indices (R2 values) of the AUC/MIC, C_max_/MIC, and %T > MIC were 0.9929, 0.9783, and 0.8371, accordingly. Based on R^2^ values, the AUC/MIC exhibited an elevated prediction of the antimicrobial effect, which showed similarity with the earlier study [35, 36]. However, using the AUC/MIC to investigate the breakpoints, the time dimension possessed by this PK-PD index makes researchers confused. Compared with the AUC/MIC, dividing the AUC/MIC by the time interval is a more universal metric that can be used not only as a scoring figure but also for computing different doses [7]. In the current study, results indicated that dividing the AUC/MIC by 24 h required for bacteriostatic action, the 1-log10 reduction, 2-log10 reduction, and a 3-log10 reduction was 0.325, 0.4375, 0.63, and 0.95, accordingly. These values were considerably lower than the values of the earlier studies that reported ENR concentration in intestinal contents of swine and broiler chicken, rather than the ENR concentration in plasma [19, 24]. Earlier studies have revealed that the ENR concentration in tissues has been increased as compared with the plasma, particularly in lung tissues, and the T1/2e of ENR in tissues was longer than that in plasma [27]. To achieve the 3-log10 reduction in vivo, a 0.95 value of dividing the AUC/MIC by 24 h needs to be achieved. It means that maintaining an average plasma concentration of ENR equal to 95% of the corresponding MIC is necessary over the dosing interval of 24 h. The reason for this result may be related to Antimicrobial Susceptibility Testing. Antimicrobial Susceptibility Testing does not take into consideration factors such as disease severity and pathogen load in the biophase [37]. Those factors may influence treatment outcomes. In the current study, to be consistent with the situation in vivo, mice plasma was used as a biological matrix to determine MIC. Despite this approach, many clinical factors, such as time of initiation relative to the development of the disease and individual differences, influence the outcome [38].

The neutropenic mouse thigh infection model is usually used for the prediction of the therapeutic dose and antibacterial effect for veterinary drugs [39]. Based on the current calculation, when the MIC_90_ was 0.25 μg/mL, the daily dose for dairy cattle to achieve -1log10 reduction and -3log10 reduction was 4.05 mg/kg and 11.1 mg/kg. For the steers, the doses were 1.42 mg/kg and 6.78 mg/kg. The reasons for the gap include the different clearance of ENR in dairy cows, steers, and the difference in plasma protein binding. The clearance of dairy cows is almost twice that of steers. The plasma proteins of dairy cows and steers are 59.4 and 60.8%. The conversion rate is very low in pigs, which was estimated to be 10% according to the previous report [22]. For pigs, the daily doses to achieve -1log10 reduction and -3log10 reduction were 0.66 mg/kg and 1.45 mg/kg. The calculated dose is similar to that calculated by Wang et al. [19]. The three MIC cut-off values needed to assist the selection of Clinical Breakpoint (CBP) comprising epidemiological cut-off values described by EUCAST as the ECOFF, PK/PD cut-off named by EUCAST as the PK/PD breakpoint, and, in veterinary medicine, a MIC cut-off related to clinical outcomes [37]. The ECOFF value of ENR against *E. coli* was 0.125 μg/mL (https://mic.eucast.org/Eucast2/SearchController/search.jsp?action=performSearch&BeginIndex=0&Micdif=mic&NumberIndex=50&Antib=-1&Specium=162). The value of selected MIC (0.25 μg/mL) covered the ECOFF. Based on the calculation, the current dose of 2.5 mg/kg can achieve the therapeutic effect for pigs, fail to achieve the therapeutic effect either for a dairy cow or for a steer. The emergence of bacterial resistance in dairy cows and steer is related to the nonstandard use of ENR in China for many years. Due to this situation, it is necessary to control drug resistance with a reasonable dose. According to published reports, fluoroquinolone resistance is steadily growing in China, with ENR resistance being the most prominent [40, 41]. Earlier studies have revealed that in Shandong province and Anhui province, the *E. coli* resistance rate for ENR was 67.5 and 56.4%, accordingly [41, 42]. While in eastern China, the rate of *E. coli* resistance to fluoroquinolone was estimated as 62.09–69.78% [43]. ENR has been used in China for several years and, in many farmlands, its usage is not regulated properly where the ranchers and farmers misuse this antibiotic. This may cause the plasmid-mediated ENR resistance to spread among bacteria, leading to the resistance against drugs [44]. Three mechanisms for plasmid-mediated fluoroquinolone resistance (PMQR) have been revealed including the Qnr (qnrA, qnrB, qnrC, qnrD, and qnrS) proteins, the aac (6′)-Ib-cr enzyme, and QepA and OqxAB plasmid-mediated efflux pumps [45, 46]. Antibiotic resistance is emerging at an alarming level in the world, and because of this problem, the PK-PD model was used to design reasonable drug doses. Several approaches such as the use of drug combinations have been recommended to significantly enhance the antibacterial effect [47]. The combined use of β-lactam and fluoroquinolones has been indicated to be effective for treating Gram-negative bacteria [48]. Their combined effect will overcome the problem of bacterial resistance. It should be noted that the theoretical values obtained from the experiment need to be verified in clinical field.

Several limitations are associated with this model. Firstly, the infection induced in the laboratory model cannot be the same as the natural infection. As a single microorganism is used for inducing infections in laboratory models, such as in the existing experimental model, only *E. coli* was used for infection. But, natural infections are mostly caused by many types of microbes. Secondly, individual differences in laboratory animals may lead to differences in results, thus cause instability. Future research should take these points into account.

## Conclusion

A neutropenic thigh infection model was employed for the evaluation of in-vivo PK and PD parameters of ENR against *E. coli*. The correlation of the PK-PD index (AUC/MIC) with the antibacterial activity was found to be the strongest. Moreover, the findings of this study showed the bacteriostatic effect, 1-log10 reduction, 2-log10 reduction, and 3-log10 reduction of the bacterial count when dividing the AUC/MIC by 24 h reached 0.325, 0.4375, 0.63, and 0.95, accordingly. Our study provides significant results about the pharmacological profile of ENR that could help in improving its clinical use against colibacillosis.

## Materials and methods

### Chemicals, organisms, and animals

Ciprofloxacin (CIP) and Enrofloxacin (ENR) with 98% purity were procured from Shanghai Aladdin Bio-Chem Technology Co., Ltd. (Shanghai, China). The solutions of antimicrobial agents (test solutions) were freshly prepared before use. The culture medium used in this experiment was purchased from Hope Biol-Technology Co. Ltd. (Qingdao, Ching). Acetonitrile was purchased from TEDIA (Fairfield, CT, USA). All reagents used in this experiment were of high-performance liquid chromatography (HPLC) grade.

The selected pathogenic *E. coli* strain (*n* = 15) was previously isolated from the dairy cow suffered from colibacillosis in the Heilongjiang Province of China. The strain was employed for the PD evaluation and developing the *E. coli* infection model. A completely characterized *E. coli* strain i.e.*,* ATCC25922 was procured from the China Institute of Veterinary Drug Control. All strains were frozen in a ^− 80^ °C refrigerator until use. To preserve bacterial culture, the bacterial cultures were sub-cultured on Mueller-Hinton agar, followed by incubation for 24 h at 37 °C.

Six weeks-old specific pathogen-free (SPF) ICR mice (female), with body weight of 30–35 g were acquired from Beijing Vital River Laboratory Animal Technology CO., Ltd. (Beijing, China). These mice were housed under SPF maintained conditions with 12 h (1:1) of light and dark cycle and sterile feeds. Mice were maintained according to the guidelines of the American Association for Accreditation of Laboratory Animal Care [13]. All animal experiment procedures were approved by the Laboratory Animal Welfare and Ethics Committee of Northeast Agricultural University (NEAUEC20190629). The study was carried out in compliance with the ARRIVE guidelines.

### In-vitro susceptibility studies

In the Heilongjiang province of China, a total of 67 clinical strains of bacteria were collected from dead dairy cows (died of colibacillosis) between 2016 and 2018. Mueller Hinton Broth (MHB) and mouse plasma were used for the MIC determination of ENR against *E. coli* via micro dilution methods, following the recommendations of Clinical and Laboratory Standards [14]. Briefly, colonies were transferred into MHB and placed on a shaking incubator at 37 °C (220 rpm) for 6 h [having reached 10^8^ CFU/mL]. A series of two-fold dilutions of drug concentration was achieved by adding 100 μL culture aliquots to a 96-well plate. The MIC was considered the lowest concentration of ENR to inhibit apparent bacterial growth in matrices after 24 h’ incubation. Susceptibility test was performed in triplicate, and *E. coli* ATCC25922 was used as a control strain. Three overlapping sets of doubling dilutions were used to improve the accuracy of the MIC determinations (0.0068–55, 0.0078–64, and 0.0088–72 μg/mL). The QC of the control strain *E. coli* ATCC25922 was 0.008–0.03 μg/mL. SPSS Ver. 16 (IBM, Armonk, NY, USA) was used for the determination of MIC_50_ to analyze the population PD of ENR against the clinical strains of *E. coli*. For the establishment of the infection model, an appropriate clinical strain of *E. coli* was selected based on the ENR distribution of MIC.

### Neutropenic mouse thigh infection model

To induce profound and sustained neutropenia (< 100 neutrophils/mm3), cyclophosphamide was injected into ICR mice (female) intraperitoneally as described previously i.e.*,* 150 mg/kg daily for 4 days and then one dose at 100 mg/kg on day 5 [15, 16]. The thigh infection model was established by a 0.1 mL inoculum injection (intramuscular), in each thigh i.e., four thighs per group per time point.

Both bacterial cultures were incubated for 24 h to achieve the exponential phase of bacterial growth. These cultures were then injected into the thigh of mice after dilution, the bacterial counts of the inoculums reached 10^6^ CFU/mL.

### Establishment of HPLC for detection of drug concentration

**Fig. 7:**
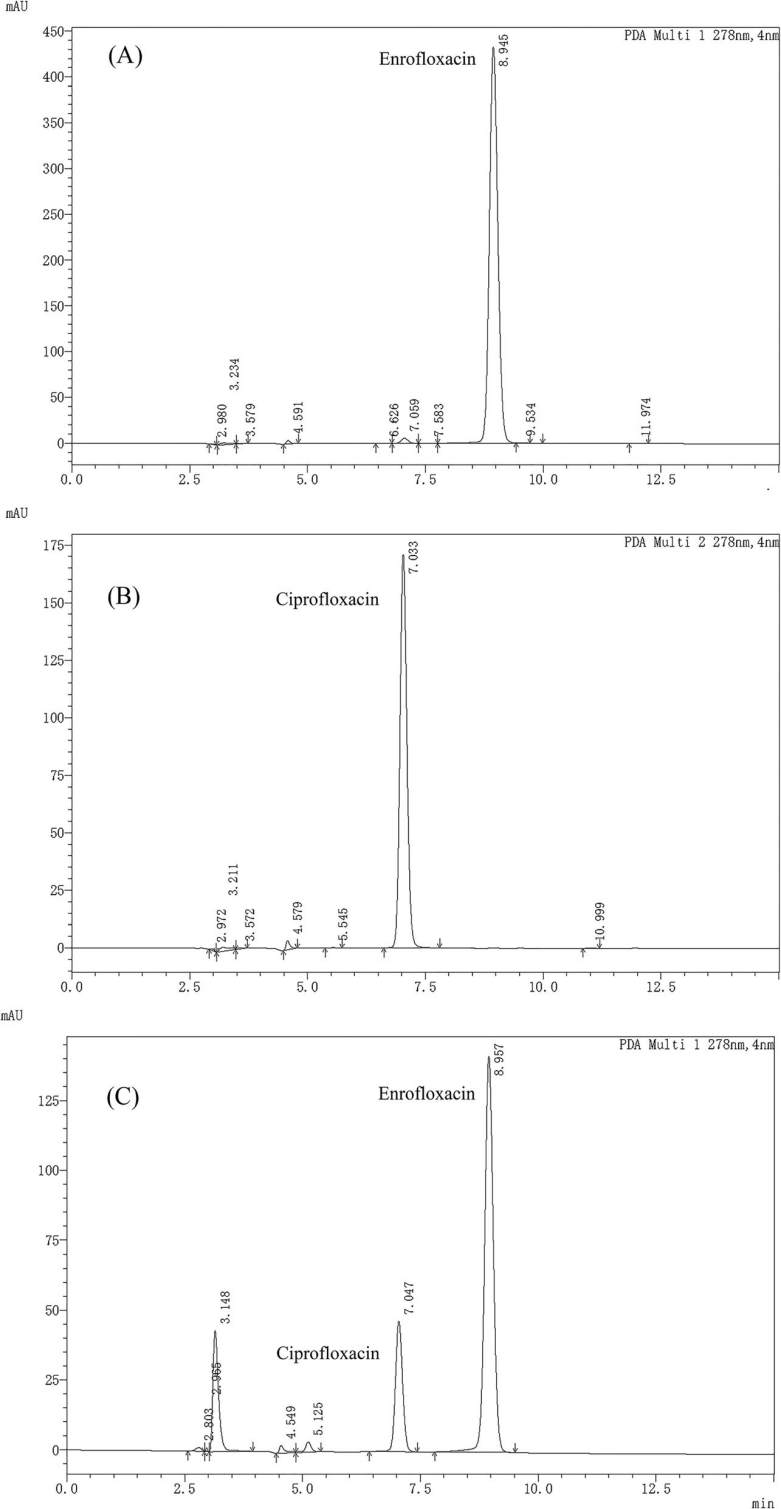
The chromatogram of standard solution and test sample. (**A**) depicts the chromatogram of standard ENR solution, (**B**) depicts the chromatogram of standard CIP solution, while (**C**) shows the chromatogram of an isolated test sample that contain ENR along with its metabolite CIP

ENR and CIP plasma concentration were determined using a Shimadzu LC-20AT series reverse-phase HPLC as described previously [17]. Briefly, 300 μL acetonitrile was combined with 100 μL plasma and the mixture was vortexed for 5 min. The mixture was then centrifuged at 7500 g for 10 min, followed by evaporating the supernatant at 60 °C under nitrogen. The residues were constituted in a 200 μL mobile phase and vortexed. From the reconstituted sample, an aliquot (20 μL) was injected into the HPLC system. To investigate the detection of ENR and CIP, the detection of PAD and spectral data were recorded at 278 nm wavelength (at ambient temperature i.e.*,* 25 °C). The retention times of ENR and CIP in plasma were found to be 9.0 ± 0.5 min and 7.0 ± 0.5 min, respectively. The retention times of both ENR and CIP in extracted plasma samples were found to be similar to the peak of respective standards, as displayed in figure (Fig. 7).

The linearity of ENR and CIP quantitation was within 0.01–5 μg/mL, while the correlation coefficient (R^2^) of both antibiotics was found to be greater than 0.99. The ENR and CIP extraction recovery from plasma was greater than 80%, while their coefficients of variation were less than 10% within runs and between runs. The detection and quantification limits were 0.005 μg/mL and 0.01 μg/mL, respectively. Winnonlin ver. 5.2.1 (Pharsight, Saint Louis, MO, USA) was employed for the evaluation of PK parameters, such as T_1/2e_ (the elimination half-life), AUC (area under plasma concentration-time curve), C_max_ (maximum concentration in plasma)_,_ and T_max_ (time of maximum plasma concentration).

### Pharmacokinetic experiment

To determine the single-dose PK parameters of ENR and CIP in neutropenic mouse thigh infection model, a 0.2 mL injection of ENR with various concentrations such as1.25, 2.5, 5.0, and 10.0 mg/kg body weight was administered. After administration of ENR, blood samples were taken via retro-orbital puncture at 0.083, 0.167, 0.25, 0.5, 0.75, 1, 2, 4, 6, 8, 10, 12, and 24 h, and were collected into 1.5 mL heparinized sodium tubes, followed by centrifugation (4000×g for 10 min at 4 °C). The plasma portion was isolated and frozen at − 80 °C until further use. The sampling of each mouse was performed at one or two time points to reduce inter-animal variability, improve PK data accuracy, and minimize the number of mice used for experiments. The underlined blood sampling strategy is relatively less stressful and closer to acceptable animal welfare. Proper management of sedation was administrated as reported earlier [18]. The mice were kept in an induction chamber with conditions of oxygen flow rate at 0.5 to 1.0 L/min. Vaporized isoflurane (3–5%) was employed as induction and was subsequently decreased (1–3%) for maintenance.

### In-vitro killing curves and in-vivo PD evaluation

To assess the anti-infective potential of the antibiotic, in-vitro bacterial killing curves were analyzed according to the previously reported method [19]. In-vitro bacterial killing curves were constructed in the MHB. An overnight incubated bacterial culture was diluted to 10^6^ CFU/mL or 10^8^ CFU/mL, followed by incubation of the high-density-pathogens and low-density-pathogens groups with 0.5 × MIC, 1 × MIC, 2 × MIC, 4 × MIC, 8 × MIC, 16 × MIC, and 32 × MIC ENR at 37 °C, accordingly. Samples were then evaluated at 1, 3, 6, 9, 12, and 24 h of incubation. At each time point, the serial dilutions of 100 μL culture were prepared in sterile saline and added to trypticase soy agar (TSA) plates. The visible bacterial colonies were counted after 12 h. The detection limit was found to be 100 CFU/mL. After the establishment of the neutropenic thigh infection model, the mice were randomized into 12 experimental groups (*n* = 5 per group). After four hours of *E. coli* Heilong 15 inoculation, these experimental groups were exposed to ENR via s.c. delivery. This was selected as the 0.0 h time point for the treatment. The doses were 1.25, 2.5, 5, 10 mg/kg at each 8, 12, and 24 h, respectively. After 24 h of ENR treatment, each mouse of the experimental group was sacrificed via CO_2_ asphyxiation. Mice allocated to the control group (untreated) were sacrificed before the 0.0 h time point for ENR treatment and 24 h post-treatment (n = 5 per timepoint). The colony counting in the homogenate of the thigh was conducted according to the previously reported method [20]. Each sample was taken from the thigh area aseptically, followed by homogenizing in 5 mL sterilized saline solution and then, 10-fold serial dilution of homogenates was prepared. One hundred microliters of all thigh homogenate dilutions were transferred into TSA media plates. Each sample was determined in triplicate. Finally, the bacterial colonies were counted on TSA media plates after a period of 12 h incubation at 37 °C, and the obtained data were analyzed via mean values. The LOD was 100 CFU/mL.

### PK-PD integration and modeling

The Inhibitory Effect Sigmoid Emax Model in the WinNonlin software (version 5.2.1, Pharsight, St. Louis, MO, USA) was used to simulate the relationship between indexes AUC/MIC, C_max_/MIC of ENR, and in vivo effectiveness.

$$ \mathrm{E}=\mathrm{Emax}-\frac{\left(\mathrm{Emax}-\mathrm{E}0\right)\times {\mathrm{C}}_{\mathrm{e}}^{\mathrm{N}}}{{\mathrm{EC}}_{50}^{\mathrm{N}}+{\mathrm{C}}_{\mathrm{e}}^{\mathrm{N}}} $$

Herein “E” depicts the antibacterial effect (determined as the alteration in bacterial counts (log CFU/thigh) in the sample 24 h post-treatment, compared with the initial number of visible colonies; E_max_ is △logCFU_24 h_ in the control sample (drug-free); E0 is △logCFU_24 h_ in the experimental sample comprising ENR when the maximum potential against bacterial growth was obtained; C_e_ is the PK-PD index (AUC/MIC, %T > MIC and C_max_/MIC for the drug concentration in plasma); EC_50_ represents the PK-PD index for the drug which shows 50% of the highest antibacterial effect; N represents Hill coefficient (demonstrating the steepness of the effect curve obtained from PK-PD index). The variance (that might be caused via regression with PK-PD indices) was estimated through the coefficient of determination.

### Dose estimation

The following formula was used to estimate dosages in different magnitudes of efficiency (1-log10 reduction and 3-log10 reduction) to deduce an optimal regimen in dairy cows, steer, and pigs [7].

$$ \mathrm{Does}=\frac{\mathrm{Clearance}\left(\mathrm{per}\ \mathrm{day}\right)\times \mathrm{factor}\times {\mathrm{MIC}}_{90}}{\mathrm{fu}\times \mathrm{F}}\div \left(1/\mathrm{conversion}\ \mathrm{rate}\right) $$

Plasma clearance is expressed in L/day. The factor is the dividing AUC/MIC by 24 h. MIC_90_ is the 90th percentile of MIC distribution. fu is the free (unbound) fraction. F is the absolute bioavailability. The clearance、fu、F and conversion rates were cited from the previous studies [21, 22].

### Statistical analyses

ANOVA (analysis of variance) was employed for the statistical evaluation of obtained data. Bonferroni’s correction was applied for the analysis of statistical significance among groups. The *P*-value < 0.05 was found to be statistically significant.

## Data Availability

The datasets used and/or analyzed during the current study are available from the corresponding author on reasonable request.

## Abbreviations

AUC: The area under the concentration-time curve
AUC/MIC: The area under the concentration-time curve to MIC ratio
CBP: Clinical breakpoint
Cmax: The peak concentration
Cmax/MIC: The peak concentration divided by the MIC
ECOFF: Epidemiological cutoff value [synonym of wild-type cutoff value (CO_WT_)]
HPLC: High-performance liquid chromatography
MBC: Minimum bactericidal concentration
MIC: Minimum inhibitory concentration
PK/PD: Pharmacokinetic/pharmacodynamic
T1/2e: Elimination half-life
Tmax: The time to achieve maximum concentration
